# A Multi-Center, Qualitative Assessment of Pediatrician and Maternal Perspectives on Rotavirus Vaccines and the Detection of *Porcine circovirus*

**DOI:** 10.1186/1471-2431-11-83

**Published:** 2011-09-26

**Authors:** Daniel C Payne, Sharon Humiston, Douglas Opel, Allison Kennedy, Mary Wikswo, Kimberly Downing, Eileen J Klein, Ana Kobayashi, David Locke, Christina Albertin, Claudia Chesley, Mary A Staat

**Affiliations:** 1 Epidemiology Branch, Division of Viral Diseases, National Center for Immunization and Respiratory Diseases, Centers for Disease Control and Prevention, Atlanta, Georgia, USA; 2 Department of Pediatrics, University of Rochester School of Medicine and Dentistry, Rochester, New York, USA; 3 Children's Mercy Hospitals and Clinics, Kansas City, Missouri, USA; 4 Department of Pediatrics, University of Washington, Seattle, Washington, USA; 5 Health Services Research and Evaluation Branch, Immunization Services Division, National Center for Immunization and Respiratory Diseases, Centers for Disease Control and Prevention, Atlanta, Georgia, USA; 6 Institute for Policy Research, University of Cincinnati, Cincinnati, Ohio, USA; 7 Department of Pediatrics, University of Cincinnati College of Medicine, Cincinnati Children's Hospital Medical Center, Cincinnati, Ohio, USA; 8 Office of the Director, Division of Viral Diseases, National Center for Immunization and Respiratory Diseases, Centers for Disease Control and Prevention, Atlanta, Georgia, USA

**Keywords:** rotavirus vaccine, RotaTeq™, Rotarix^®^, porcine circovirus (PCV), adventitious virus, pediatricians, communication development, focus groups

## Abstract

**Background:**

In 2010, researchers using novel laboratory techniques found that US-licensed rotavirus vaccines contain DNA or DNA fragments from *Porcine circovirus *(PCV), a virus common among pigs but not believed to cause illness in humans. We sought to understand pediatricians' and mothers' perspectives on this finding.

**Methods:**

We conducted three iterations of focus groups for pediatricians and non-vaccine hesitant mothers in Seattle, WA, Cincinnati, OH, and Rochester, NY. Focus groups explored perceptions of rotavirus disease, rotavirus vaccination, and attitudes about the detection of PCV material in rotavirus vaccines.

**Results:**

Pediatricians understood firsthand the success of rotavirus vaccines in preventing severe acute gastroenteritis among infants and young children. They measured this benefit against the theoretical risk of DNA material from PCV in rotavirus vaccines, determining overall that the PCV finding was of no clinical significance. Particularly influential was the realization that the large, randomized clinical trials that found both vaccines to be highly effective and safe were conducted with DNA material from PCV already in the vaccines.

Most mothers supported the ideal of full disclosure regarding vaccination risks and benefits. However, with a scientific topic of this complexity, simplified information regarding PCV material in rotavirus vaccines seemed frightening and suspicious, and detailed information was frequently overwhelming. Mothers often remarked that if they did not understand a medical or technical topic regarding their child's health, they relied on their pediatrician's guidance.

Many mothers and pediatricians were also concerned that persons who abstain from pork consumption for religious or personal reasons may have unsubstantiated fears of the PCV finding.

**Conclusions:**

Pediatricians considered the detection of DNA material from PCV in rotavirus vaccines a "non-issue" and reported little hesitation in continuing to recommend the vaccines. Mothers desired transparency, but ultimately trusted their pediatrician's recommendation. Both vaccines are currently approved for their intended use, and no risk of human PCV illness has been reported. Communicating this topic to pediatricians and mothers requires sensitivity to a broad range of technical understanding and personal concerns.

## Background

Rotavirus vaccines are oral, live, attenuated vaccines that have been shown in large clinical trials and through post-licensure surveillance to be safe and highly effective in protecting children against severe rotavirus gastroenteritis [1-8]. During the course of testing novel virus-detection techniques, Victoria et al. [9] unexpectedly identified nucleic acids from one adventitious virus in Rotarix^®^, the licensed, monovalent rotavirus vaccine produced by GlaxoSmithKline Biologicals (Rixensart, Belgium). The detected virus shared 98% identity to *Porcine circovirus *(PCV) -*1*, and covered the complete circular genome. PCV is a virus that commonly infects pigs and has been detected in 5% of stool samples from US adults, likely as a result of dietary consumption of pork products [9], but PCV is not believed to cause illness among humans [9-11].

In early 2010, this finding was reported to the US Food and Drug Administration (FDA). On March 22, 2010, FDA released a recommendation for clinicians to temporarily suspend use of Rotarix^®^, although there was no evidence of a safety risk. On May 6, 2010, FDA reported preliminary findings that RotaTeq™, the licensed, pentavalent rotavirus vaccine produced by Merck and Co. (Whitehouse Station, NJ), also contains detectable PCV (-*1 and -2*) material. On May 7, 2010, the FDA Vaccines and Related Biological Products Advisory Committee (VRBPAC) convened. On the basis of available evidence regarding a theoretical risk of PCV infection among humans and the observed benefits of rotavirus vaccines in preventing severe acute gastroenteritis among infants, VRBPAC panelists expressed reassurance that the detection of DNA and DNA fragments from PCV in rotavirus vaccines was not likely to cause harm to humans and recommended that information on this topic be provided prior to vaccination.

On May 14, 2010, FDA issued a recommendation for clinicians to resume use of Rotarix^® ^and to continue use of RotaTeq™. Subsequent investigation suggests that PCV material was introduced into both rotavirus vaccines through porcine-derived trypsin--a reagent used in the cell-culture growth process of vaccine production--commencing several years before randomized, controlled, multi-center phase III clinical vaccine trials were conducted for both vaccines [12,13].

Previous findings of adventitious viruses in live, attenuated vaccines have been relatively rare and followed by studies determining the agents to not be associated with human illness [14-16]. These instances have often arisen through inadvertent introductions of adventitious viruses or viral materials during vaccine production, often via incomplete inactivation during the attenuation process.

In June 2010, the US Centers for Disease Control and Prevention (CDC) partnered with three existing rotavirus surveillance sites to conduct focus groups of pediatricians and mothers to assess their perspectives and the communications issues regarding rotavirus vaccines and PCV. An assessment to measure whether focus group participants changed their attitudes regarding rotavirus vaccines also followed the focus group discussions.

## Methods

### Interview Procedures

We conducted three iterations of focus groups for pediatricians and mothers in Seattle, WA; Cincinnati, OH; and Rochester, NY (total of 9 physician and 9 parent focus groups). A standard protocol, moderator guides, and pre-/post- test comparisons of acceptability of the rotavirus vaccine were uniformly applied.

The initial focus groups were held in July 2010, approximately 4 months following the first public announcement of PCV in a rotavirus vaccine, and approximately 2 months following the FDA recommendation to resume/continue rotavirus vaccinations. Two further focus group iterations occurred in August and September 2010. All focus group iterations included core discussions on rotavirus disease and rotavirus vaccines, followed by information on PCV. Co-investigators developed communication materials and messages prior to the focus group sessions and obtained parent and pediatrician feedback on these materials during the focus group discussions. The moderator's guide and communication materials were then revised iteratively based upon comments and data obtained from the previous focus groups until conceptual insights were exhausted. The focus groups lasted approximately 90 minutes. Although each moderator was independent and experienced in leading focus groups, participant responses may be affected by the manner in which information is conveyed to the focus groups.

### Study Population

Each focus group consisted of up to 8 unique participants. Physician participation was restricted to actively practicing, board-certified/-eligible, non-military, primary care pediatricians who typically administered >4 rotavirus vaccine doses per week. In each focus group, only one pediatrician participated from any given office. Participants were excluded if the pediatrician or his/her immediate family member worked in vaccine development, marketing, research, or regulation. Recruitment of pediatricians occurred through community pediatrician list serves, at events (e.g., grand rounds), and other administrative meetings, and the study team followed up with interested participants for a determination of eligibility.

Mothers at least18 years of age who reported having normally developing children between 6 months and 4 years of age were eligible for participation if they 1) were fluent and literate in English, 2) agreed to routine vaccination for the index child, and 3) at least partially influenced health-care decisions for their child. The ineligibility of mothers with an infant <6 months of age was intended to avoid the focus group discussions from influencing a mother's decision to have a current infant vaccinated or not. Potential participants were excluded if they or any immediate family member worked in vaccine development, marketing, research, or regulation; if they participated in any market study within the previous 6 months; or if they worked in the healthcare field. For each focus group, recruiters attempted to enroll at least 2 mothers with the educational attainment of a high school diploma or less. While these eligibility and exclusionary criteria for mothers were consistently applied across participating sites, the sampling frame and solicitation methods for mothers differed by site. In Seattle, mothers were recruited from a university-based primary care clinic, but in Cincinnati and Rochester a marketing telephone list was used to contact households for eligibility.

### Interview Guide

Focus group questions were open-ended, non-sensitive, and designed to maintain participant privacy. They included the following domains: a) perceptions of rotavirus disease, b) perceptions of rotavirus vaccination, c) attitudes toward the detection of PCV material in rotavirus vaccines, and, d) attitudes toward communication materials on these topics.

### Qualitative Data Analysis and Presentation

After each round of focus groups at each site, the audiotaped discussions were transcribed verbatim and then analyzed by use of an inductive coding technique for qualitative data [17,18]. Each investigator independently read each focus group transcripts specific to their site(s) and abstracted key themes regarding rotavirus disease, rotavirus vaccine, attitudes regarding the PCV finding, and communications. Investigators then met to discuss their independent analyses and negotiate a final list of themes. Quotations used in this article were excerpted from transcripts and are representative of the category to which they have been assigned. Group dynamics inherently affect focus group results and this was taken into consideration during our analysis.

### Comparison of Participants' Pre- and Post-Focus Group Questionnaires

We asked attitudinal questions at the beginning and end of each focus group to compare whether or not the focus group discussion had influenced or changed subject perceptions of rotavirus vaccines. These questions included attitudes and opinions regarding rotavirus vaccination (i.e., their acceptability, understanding and barriers to rotavirus vaccination). Changes in pre- and post-focus group questionnaire responses were assessed using the Wilcoxon signed rank test for non-normal distributions. For these comparisons, the estimated aggregated study power was >90% for pediatricians and mothers.

### Ethics

Prior to focus group enrollment, the CDC Human Research Protection Office determined this evaluation to constitute a public health non-research project, and the institutional review boards at each participating institution (Seattle Children's Hospital, University of Rochester, New York, School of Medicine and Dentistry, Cincinnati Children's Hospital and Medical Center) declared the project to be exempt. Potential participants consented to participate at screening and if they decided not to participate, they were thanked for their time and not contacted further. Permission to audiotape was obtained during screening and again at the outset of the group.

## Results

Focus groups commenced approximately 4 months following the first public announcement of PCV in a rotavirus vaccine and approximately 2 months following the FDA recommendation to resume/continue rotavirus vaccinations. Results are presented in order of characteristics of the subjects, major respondent themes (pediatrician, parent, overarching), communication recommendations, and results of the pre-/post-focus group comparison of attitudes and opinions regarding rotavirus vaccines.

### Participant Characteristics

We conducted focus groups in three different regions of the United States (the West, Midwest, and Northeast). Table 1 describes the characteristics of pediatricians (n = 45) (Table 1) and mothers (n = 58) (Table 2) participating in focus groups. Pediatricians had an average 15.4 years of experience (median = 15, range = 1-39) following residency, and most (62%) belonged to a private practice group. About one-third of the children enrolled by their pediatric offices were publicly insured and eligible for the Vaccines for Children Program, [19] a federally-funded entitlement program providing vaccines at no cost to socioeconomically disadvantaged children. Most pediatricians solely administered RotaTeq™ (76%), compared with 4% who solely used Rotarix^® ^and 20% who reported using both vaccines.

**Table 1:** Characteristics of pediatricians participating in focus groups by site.

Pediatrician Characteristics	Seattle (N = 12)	Rochester (N = 16)	Cincinnati (N = 17)	Total (N = 45)
**Average number of years since residency (years)**	10.2	15.6	18.0	15.4 range (1-39)

**Description of practice**				
Solo	0	4	0	4
Private group	8	9	11	28
Non-profit community	1	0	4	5
Medical school-based practice	2	1	2	5
Hospital-based practice	1	1	0	2
Other	0	1	0	1

**Average number of full-time physicians in practice**	23.7	3.9	7.9	10.7 range (1-75)
**Location of practice**				
Urban	9	4	7	20
Suburban	3	11	10	24

**Average number of newborns (<1 month old) enrolled in practice last month**	77.9	22.3	39.7	43.7 range (3-300)

**Average percentage of children with public insurance/Medicaid in practice**	27%	27%	40%	32%

**Average percentage of children eligible for Vaccines For Children (VFC) in practice**	35%	31%	41%	36%

**Which rotavirus vaccine do you use?**				
RotaTeq™	12	12	10	34
Rotarix^®^	0	1	1	2
Both	0	3	6	9
Neither	0	0	0	0

**Before rotavirus vaccine was available, rotavirus was the most common cause of severe infectious diarrhea in children <2 years old in the U.S**.				
(% agreed)				
Strongly agree (1)	5	9	12	26
Somewhat agree (2)	4	3	4	11
Somewhat disagree (3)	2	1	1	4
Strongly disagree (4)	1	3	0	4
Average score	1.9	1.9	1.4	1.7

**Before rotavirus vaccine was available, rotavirus was responsible for more hospitalizations annually than influenza**.				
(% agreed)				
Strongly agree (1)	2	6	6	14
Somewhat agree (2)	5	5	10	20
Somewhat disagree (3)	5	4	1	10
Strongly disagree (4)	0	1	0	1
Average score	2.3	2.0	1.7	2.0

**Table 2:** Characteristics of mothers participating in focus groups by site.

Parental Characteristics	Seattle (N = 13)	Rochester (N = 18)	Cincinnati (N = 27)	Total (N = 58)
**Average maternal age (years)**	34.5	31.3	32.9	33.2 range (21-44)

**Level of maternal education**				
Less than high school	1	2	0	3
High school	2	4	5	11
Some college	1	6	10	17
College	2	5	9	16
Post-college graduate degree	7	1	3	11

**Location of household**				
Urban	10	8	2	20
Suburban	1	10	22	33
Rural	1	0	3	4

**Insurance status**				
Public insurance/Medicaid	4	11	4	19
Private	8	7	20	35
Uninsured	0	0	1	1
Other	0	0	2	2

**Average number of children in household**	2.5	2.3	2.2	2.3

**Have heard of rotavirus vaccine before (%)**	83%	56%	93%	79%

More than 80% of pediatricians from all sites either strongly or somewhat agreed to the statement, "Before rotavirus vaccine was available, rotavirus was the most common cause of severe infectious diarrhea in children <2 years old in the US." About three-quarters (76%) strongly or somewhat agreed that rotavirus was responsible for more annual hospitalizations than influenza during the pre-vaccine era.

The average age for participating mothers was 33.2 years (median = 33.5, range = 21-44), and 76% had achieved more than a high school education. Most mothers (61%) reported being privately insured, compared with 33% having public insurance/Medicaid. The average household had 2.3 children, and most mothers (79%) had heard of rotavirus before the focus group.

### Pediatrician Themes

Many pediatricians had negative memories of rotavirus infections and had observed a high burden of childhood disease from rotavirus gastroenteritis, as represented by common remarks included in Table 3. Those pediatricians who had the opportunity to observe rotavirus both during and after the pre-vaccine era understood firsthand the success of rotavirus vaccines in preventing severe pediatric acute gastroenteritis (Table 4).

**Table 3:** Examples of common comments from pediatricians regarding rotavirus disease.

"We have lots of experience with rotavirus disease. I think vomiting and diarrhea ranks up there with my least favorite illness, because it's a lot of supportive care, a lot of phone calls, a lot of coming back to the office, a lot of reevaluation, and there's nothing you can do other than fluids."
"Most kids did okay on oral rehydration, but we had a lot who were hospitalized for IV therapy."

"I went through residency in the late '80 s, and we used to have an entire ward that would be filled with kids with really horrible diarrhea. Entire wards that would fill up the hospital, to the point that people would say, 'You don't want to be on [the infant floor] during rotaviral season.' Because ...you would get 25, 30 admissions a night and most of them would be having diarrhea and a lot of them would be almost in shock because of it. You could see how in a place where there weren't resources for IV fluids that there would be a lot of deaths from dehydration."

"I remember how contagious it is, and how it lives on surfaces and we'd have to gown, glove, mask, and practically use dedicated equipment for the rooms in hospital. And if you suspected it in the outpatient setting, you had to be extremely careful by cleaning the room down. It just spread within families, but the babies would be the sickest."

"Over the years, we've had some significantly ill kids from [rotavirus]. One kid had a shock-like response to rotavirus and it totally devastated him, so that he's probably one of the sickest little developmentally devastated kids in the practice and it was from rotavirus years ago."

**Table 4:** Examples of comments from pediatricians regarding the post-licensure rotavirus vaccination period.

"It's been interesting to see over the years. [Before the vaccine was introduced] you would get kids coming in just bone dry, couldn't find the vein to start an IV... And to see that all change so much, it's been really remarkable."
"Well it's definitely changed over time since the advent of the vaccine. It wasn't that uncommon to have to send kids over [to be hospitalized] for rehydration or to work through the severe diarrhea and the vomiting. [Now] it's just not as common at all to have to refer for that."

"...after [rotavirus vaccine] was introduced, it was nice to see the drastic change. ...maybe [hospitalizations for IV therapy] dropped by 90 percent."

Overall, pediatricians reported that the mothers of their patients rarely hesitated to accept rotavirus vaccination. Specifically noted were reports that, "[*Mothers] are willing to accept [rotavirus vaccine] when you say the virus gives horrible vomiting and diarrhea and it can last two weeks." *As the vaccines are orally administered, pediatrician participants stated that many mothers do not count it among the injections recommended in the vaccination schedule.

At the time of the focus groups, most participating pediatricians had some prior knowledge that DNA material from PCV had been detected in rotavirus vaccines. When given detailed information on this topic, many seemed to intuitively measure the theoretical risks of PCV against the benefits they personally observed from rotavirus vaccination. Nearly every participating pediatrician expressed an opinion that the detection of DNA or DNA fragments from PCV in rotavirus vaccines was not clinically important, repeatedly using the term "*non-issue*." Particularly influential to this appraisal was that the published, large phase III randomized clinical trials that found both vaccines to be highly effective and safe were actually conducted with DNA material from PCV already in both vaccines. Respondents reported that, *"Now I've learned that the particles have been there from day one, so if you believe the safety data on the vaccine itself then you believe that [the vaccine] is safe." *Furthermore, many pediatricians found reassuring that PCV is not known to cause illness in humans, stating, *"... [humans are] literally exposed to thousands of viruses every day and don't become ill." *Since PCV material is likely consumed by many people in pork products without adverse consequences, many observed that, *"...we are exposed to [PCV] in the pork we eat, and we're fine. So this is not our only exposure. It's not a new virus to us."*

Given their general dismissal of any theoretical risk from DNA material from PCV in rotavirus vaccines, many pediatricians indicated that they would not devote time to explain the PCV finding, although many agreed that they would provide information to mothers who specifically queried them on the topic. Their general reluctance was rooted in three stated reasons: 1) DNA material from PCV in rotavirus vaccines did not constitute any risk, so the point was both moot and unduly alarming to the mothers, 2) discussion would be lengthy (and potentially non-reimbursable) and would compete with discussion of many other important clinically relevant topics, and, 3) many mothers would not fully understand the issue even with further elaboration.

### Maternal Themes

While more than three-quarters of the participating mothers had at least some college education, many expressed a general lack of scientific and technical understanding of viruses, DNA, how vaccines are manufactured, and how vaccines work. Approximately one-fifth (21%) of the mothers reported unfamiliarity with the disease "rotavirus" and nearly none had prior knowledge of the PCV finding. One mother remarked, *"I guess what all of this brings up for me is that I don't really understand what's in vaccines... it makes me feel like I want to understand more what a vaccine actually is, that this is what's happening... that there's a virus that somehow got in there that people didn't know about." *Simplified information regarding PCV material in rotavirus vaccines seemed frightening, while detailed information was frequently overwhelming. *"I don't know what the porcine circovirus is, so to me, this statement sounds like there's another virus present in the rotavirus vaccine. So, that would concern me." *Stating that the vaccine was simply "safe" without providing evidence often brought further suspicion, exemplified by the comment, *"When they say something is safe... well, it's the classic hedge. They don't have any evidence that it's not safe, which just means that they may not have studied it enough."*

Mothers largely supported the ideal of transparency in being provided information regarding all vaccine risks and benefits. However, mothers often remarked that if they did not understand a medical or technical topic regarding their children's health they commonly relied on their pediatrician's guidance. Nonetheless, a few mothers mentioned that the detection of PCV material in rotavirus vaccines eroded their confidence in the vaccines (Table 5).

**Table 5:** Examples of comments from mothers regarding PCV materials found in rotavirus vaccines.

"I'd really like to see information [on PCV] if it was going in my child."
"Clarification would be good as to how it got there, or what purpose it serves in the vaccine. I think that would be a good thing to know."

"If my doctor is going to recommend it, more than likely, I'm just going to go with it. If they feel it's necessary, I trust them."

"I'm probably putting too much trust in my pediatrician, but I feel like he's going to tell me everything important that I need to know beforehand."

"You know, I have my 2 children and can talk about what's happened with them but, the doctor sees 20, 30, 40 kids a day so there's a lot more exposure to kids who've had the rotavirus vaccine and what happens, and what happens when they don't [get the vaccine]."

"I'm sure with all the new technology, you're going to find all kinds of things in vaccines, and as long as they're safe, I mean, if the doctor is going to recommend it, or the CDC, I think that's good enough."

"I still feel like the benefits outweigh the risks but every time I [hear] something like this, I feel a little bit less confident."

### Overarching Themes

Many mothers and pediatricians expressed alarm that those who abstain from pork consumption for religious or personal reasons may have unsubstantiated fears that PCV is pig material (rather than the fact that PCV is a virus commonly found in pigs). Concern was most directed at whether parents of Jewish or Muslim faiths would reject the vaccines due to this misunderstanding. Some participants were similarly concerned that, due to its name, *Porcine circovirus *was somehow related to H1N1 influenza (a.k.a. swine flu) or illnesses of animal origin (e.g., bovine spongiform encephalopathy, a.k.a. mad cow disease) (Table 6).

**Table 6:** Examples of comments from pediatricians representing fears and concerns regarding PCV materials in rotavirus vaccines.

"I assume you'll have people asking if it's related to swine flu if you write the name out like that. Doesn't 'porcine' mean, 'related to pigs'? Everybody's obsessed with swine flu right now, so that always worries people."
"...there are a lot of things that start within the animal population that transfer over to humans and can be far more devastating."

"Then I started thinking about the 'what-ifs' that [my patients' parents] will be asking; 'Well about that avian thing, the avian flu? It never caused a problem in anybody else, but then it found a way to combine with a human type virus."

"A lot of my [patients'] parents don't eat pork. They're very naturalist [sic]. They're going to see pork, and then they'll see virus. And they're going to stop there, and they're going to say I don't want my child to get this vaccine. They'll assume there's another virus in it, and they'll think of swine flu, pork, and then it just becomes this whole association that they'll go crazy with."

Pediatricians and some mothers cautioned that using the same acronym, PCV, for both *Porcine circovirus *and pneumococcal conjugate vaccine could cause confusion.

### Communication Recommendations

#### Vaccine Information Sheet (VIS) recommendations

Focus group participants indicated that a general statement should not replace further discussion between pediatrician and mother regarding the PCV finding. However, on the basis of themes elicited from focus group participants, the following paragraph was developed for the rotavirus vaccine information sheet (VIS) that is provided to all vaccinees:

*"A virus (or parts of a virus) called porcine circovirus (PCV) is present in both rotavirus vaccines. There is no evidence that PCV is a safety risk or causes illness in humans, and these rotavirus vaccines have been shown to be safe and effective at preventing severe diarrhea. If you have questions, ask your doctor or visit *http://www.cdc.gov/vaccines/vpd-vac/rotavirus.*"*

Nearly all pediatricians and mothers agreed that viewing VIS sheets for the first time during the office visit was inopportune. Some mothers acknowledged that the VIS was infrequently read at the health care visit, commonly stating, *"By the time your kid actually gets the shot, you've waited in the waiting room, you've waited in the greeting room. You might have one, two, or three more older kids with you. They're all melting down, and of course, when they get their shots, then that's just horrible for everybody. So, how much of this [VIS] you might actually read right there, when you need to know it before you make a decision... not going to happen." *Instead, being given this information in advance of the visit could provide mothers an opportunity to discuss the information with other caregivers and to research any concerns in advance of vaccination. Pediatricians generally remarked that they have limited time to discuss the vaccines or vaccine concerns at a well-child visit. Some pediatricians remarked that they proactively give VIS sheets as early as during the routine neonatal visits in order for parents to review future vaccinations in advance.

#### General communication perspectives

Mothers most frequently trusted the recommendations of their pediatricians. Pediatricians stated that they most trusted statements and medical alerts coming from the American Academy of Pediatrics (AAP).

Participants noted that websites of public health agencies and medical organizations should reflect relevant and updated information. Mothers emphasized that they should be referred to a specific webpage address with this information, not to a general agency or organizational home web address, to avoid difficulty in finding the correct material.

Several pediatricians using electronic medical records and automated information systems suggested that a few statements on the PCV finding be proposed that can be included in "dot phrase" information dissemination. "Dot phrases" are pieces of information that the health care provider can select to include in personalized information that can be provided during an office visit.

### Results of Pre-/Post-test

Following the focus group discussion of rotavirus, rotavirus vaccines and PCV, pediatricians showed no statistically significant changes in their recommendation of rotavirus vaccines or in perceived barriers to vaccination. Nearly all pediatricians (98%) reported no change in their opinion to recommend the vaccines (P = 1.00). Also, no appreciable net change was observed among pediatricians who reported that parental concern with vaccine safety would be a barrier to rotavirus vaccination (26% reported vaccine safety to be an increased barrier, whereas 23% reported it to be a diminished barrier; P = 0.75). (Tables 7 and 8)

**Table 7:** Pre-focus group assessment results: Pediatricians and Mothers

Initial Pediatrician Perceptions					
	**What are your current recommendations on rotavirus vaccinations for infants?**	**Perceived Barrier** **-** **Lack of insurance coverage**	**Perceived Barrier** **-** **Lack of reimbursement**	**Perceived Barrier** **-** **Mother's concern with vaccine safety**	**Perceived Barrier** **-** **Time needed to discuss with mothers**

Routinely recommend (1)	44 (98%)	3 (7%)	1 (2%)	6 (14%)	0 (0%)

Occasionally recommend (2)	0 (0%)	2 (4%)	5 (11%)	23 (53%)	2 (4%)

Inform but don't recommend (3)	0 (0%)	15 (33%)	12 (27%)	8 (18%)	23 (51%)

Recommend against (4)	1 (2%)	25 (56%)	27 (60%)	7 (16%)	20 (44%)

Average score	1.1	3.4	3.4	2.4	3.4

					

**Initial Maternal Perceptions**					

	**Rotavirus vaccine is an important vaccine for my child**	**I feel confident that rotavirus vaccine works to prevent serious diarrhea**	**I feel confident that rotavirus vaccine is safe**	**If I had another baby, I would have that baby get rotavirus vaccine**	

Strongly agree (1)	27 (53%)	20 (39%)	23 (45%)	37 (71%)	

Somewhat agree (2)	22 (43%)	30 (58%)	23 (45%)	11 (21%)	

Somewhat disagree (3)	2 (4%)	1 (2%)	5 (10%)	4 (8%)	

Strongly disagree (4)	0 (0%)	1 (2%)	0 (0%)	0 (0%)	

Average score	1.5	1.7	1.6	1.4	

**Table 8:** Comparison of Pre- and Post-focus group questionnaires: Pediatricians and Mothers

Change in Pediatrician Perceptions					
	**What are your current recommendations on rotavirus vaccinations for infants?**	**Important barrier -** **Lack of insurance coverage**	**Important barrier -** **Lack of reimbursement**	**Important barrier -** **Mother's concern** **with vaccine safety**	**Important barrier -** **Time needed to discuss with mothers**

More likely to recommend	1 (2%)	11 (25%)	5 (12%)	11 (26%)	11 (24%)

Same answer	43 (98%)	25 (57%)	32 (76%)	22 (52%)	27 (60%)

Less likely to recommend	0 (0%)	8 (18%)	5 (12%)	10 (23%)	7 (16%)

P-value *Ж*	P = 1.00	P = 0.18	P = 1.00	P = 0.75	P = 0.18

					

**Change in Maternal Perceptions**					

	**Rotavirus vaccine is an important vaccine for my child**	**I feel confident that rotavirus vaccine works to prevent serious diarrhea**	**I feel confident that rotavirus vaccine is safe**	**If I had another baby, I would have that baby get rotavirus vaccine**	

More likely to agree with statement	5 (10%)	10 (19%)	6 (12%)	1 (2%)	

Same answer	32 (63%)	37 (71%)	26 (51%)	35 (67%)	

More likely to disagree with statement	14 (28%)	5 (10%)	19 (37%)	16 (31%)	

P-value *Ж*	P = 0.01 §	P = 0.17	P < 0.01 §	P < 0.01 §	

*Ж *Wilcoxon signed rank sum test

§ indicates that the post-focus group response changed with statistical significance

Mothers, however, had more negative perceptions regarding the importance and safety of the vaccines and whether a subsequent baby would receive rotavirus vaccines. Roughly half of the participating mothers continued to feel confident that rotavirus vaccines are safe, while 37% changed their opinion to disagree with this statement. (P < 0.01) Following the focus group discussion, 16 of 52 mothers somewhat or strongly disagreed with the statement "If I had another baby, I would have that baby get rotavirus vaccine.", compared with 4 of 52 mothers stating this during the pre-focus group questionnaire. This indicates that 12 (23%) of 52 participating mothers changed their opinion on this issue following the focus group discussion. (P < 0.01) No significant change was observed regarding parental confidence that rotavirus vaccine prevents severe diarrhea (Tables 7 and 8).

## Discussion

Stating that there was no evidence that DNA or DNA fragments from PCV in rotavirus vaccines would cause harm to humans, FDA issued a recommendation for health care workers to resume administering Rotarix^® ^and to continue administering RotaTeq™. At that time, little understanding existed for how best to communicate this complicated finding. Since the inception of our qualitative study, FDA has announced that it is investigating additional testing and technologies to be implemented into the vaccine production process to prevent adventitious agents from being introduced to vaccines [20]. Additionally, both rotavirus vaccine manufacturers have changed their vaccines' labels to notify consumers of the PCV findings [21,22] (Table 9). Human risk of PCV illness has not been detected in clinical assessments [12,13], but subsequent laboratory analyses of Rotarix^® ^have detected the presence of infectious PCV-1 in a cell culture assay, and the presence of PCV-1 and PCV-2 DNA in RotaTeq™ which tested negative for growth in cell culture assays [23].

**Table 9:** Label changes to Rotarix^® ^and RotaTeq™ vaccines (circa 2010).

Rotarix^® ^label	"In the manufacturing process, porcine-derived materials are used. Porcine circovirus type 1 (PCV-1) is present in Rotarix. PCV-1 is not known to cause disease in humans."
**RotaTeq™ label**	"In the manufacturing process for RotaTeq, a porcine-derived material is used. DNA from porcine circoviruses (PCV) 1 and 2 has been detected in RotaTeq. PCV-1 and PCV-2 are not known to cause disease in humans."

Our results demonstrate that pediatricians measured the observed benefit of rotavirus vaccines against the detection of DNA material from PCV in rotavirus vaccines and concluded, overall, that this finding was not clinically significant. Nearly every pediatrician participating in our focus groups continued to report routinely recommending rotavirus vaccines to infants. Particularly influential to their appraisal was that the large, randomized clinical trials that found both vaccines to be highly effective and safe were conducted with DNA or DNA fragments from PCV already in the vaccines.

Mothers consistently supported the ideal of transparency and disclosure regarding all vaccination risks and benefits. However, the desire for informational transparency was frequently incompatible with limitations in the comprehension of core scientific and technical information exhibited by many mothers. Through testing several iterations of messages of varying sophistication and depth, we found that even providing simplified messages was often insufficient to prevent confusion over the complicated underlying concepts. Short, simple statements given to our focus group mothers on this topic often seemed alarming and potentially motivated future vaccine refusal, while providing more detailed information was considered overwhelming and provoked suspicion. However, as observed by the National Immunization Survey, in which mothers who expressed misconceptions regarding risks and benefits of vaccination were positively influenced towards vaccination through the guidance of their health care provider, [24] our results indicated that mothers commonly relied on their pediatricians' advice when confronted with a difficult medical or technical topic regarding their child's health.

In the end, building a maternal cognitive assessment of benefits and theoretical risks regarding the detection of DNA material from PCV in rotavirus vaccines is unlikely to be cultivated by simply providing an untailored textual statement. A skillfully navigated, personalized discussion between an interested mother and pediatrician, employing sensitivity to a mother's specific technical understanding and personal concerns on this topic, is likely the best option, although perhaps the most difficult one. One pediatrician offered the following scenario, *"I might ask the parents, 'Is there anything specific that you're concerned about?' and see if this comes up. If they said, 'I heard something about something in the vaccine', then I might say, 'Well, yes, we've heard of that as well, but we are not concerned." *Other pediatricians indicated that they would gauge the mother's own level of scientific understanding in discussing the topic, while still others openly acknowledged that they would avoid such a discussion altogether. If individualized information is to be shared with the mother, this should ideally occur well in advance of the vaccination event. Internet and email communications from trusted sources, such as AAP, appear effective in communicating this topic to pediatricians.

In an analysis of HealthStyles survey data by Gust et al. [25], mothers who disagreed that they had sufficient information to make a decision on whether to vaccinate a child also reported that they had less confidence in the safety of childhood vaccines and had difficulty communicating with their health care provider. Furthermore, as our data also indicate, Gust et al. suggested that a health care provider simply communicating the risks and benefits of vaccination works to build the underlying confidence in the vaccines themselves. Other studies have demonstrated that parents consistently cite their child's provider as an important and trusted influence in their immunization decision-making [24,26-29].

There are several potential limitations to this study. First, the focus group setting is an artificial environment for receiving the concentrated information that was disseminated to our participants. This may have caused participants to respond with greater intensity and suspicion regarding why the focus group was being held than they normally would have on the basis of a cursory, quotidian discussion. Our focus group participants may not be representative of the larger population; thus, the themes identified may not be generalizable. We focused upon pediatrician and maternal perspectives for this assessment but recognize the role of other health professionals in communicating this information, and fathers/other caretakers in health care decision-making. Last, as we conducted the focus groups within a brief period following the first publicly disseminated information regarding this topic, it is unclear to what extent our focus group results represent time-limited responses. Further investigation could explore psychological and media theories to speculate upon the possible permanence of these findings.

## Conclusions

We found that pediatricians did not perceive the finding of DNA or DNA fragments from PCV in rotavirus vaccines to be of clinical importance and reported near-unanimous confidence in continuing to administer rotavirus vaccines. This finding did not match the perceptions of mothers who desired to but, in many cases, had difficulty in independently assessing the potential risk-benefit profile of the rotavirus vaccines. Fostering skillful, personalized communication between pediatricians and mothers appears paramount in understanding such risk-benefit assessments regarding rotavirus vaccines.

## Competing interests

Daniel C. Payne, PhD, MSPH No competing interests to declare

Sharon Humiston, MD, MPH Past rotavirus manufacturer speaking engagement.

Douglas Opel, MD, MPH No competing interests to declare

Allison Kennedy, MPH No competing interests to declare

Mary Wikswo, MPH No competing interests to declare

Kimberly Downing, PhD No competing interests to declare

Eileen J. Klein, MD, MPH No competing interests to declare

Ana Kobayashi, MPH No competing interests to declare

David Locke No competing interests to declare

Christina Albertin, MPH No competing interests to declare

Claudia Chesley No competing interests to declare

Mary A. Staat, MD, MPH Past research funding from Merck Research Laboratories, Inc. and current funding from GlaxoSmithKline, Inc; Rotavirus Advisory Board for Merck and Company and GlaxoSmithKline, Inc.

## Authors' contributions

DP, SH, EK, and MS conceived the project, and DP, SH, DO, AKe, MW, KD, EK, AKo, CC, and MS contributed to its design. KD, AKo, CA, DL and CC implemented and oversaw the methodology. DP, SH, DO, AKe, MW, KD, EK, AKo, CC, and MS interpreted the data, MW entered and formatted the data, and DP, SH, DO and MS contributed to the principal drafting of the manuscript. All authors read and approved the final manuscript.

Disclaimer: The findings and conclusions in this report are those of the authors and do not necessarily represent the views of the US Centers for Disease Control and Prevention.

## Abbreviations Used

ACIP: US Advisory Committee on Immunization Practices; CDC: US Centers for Disease Control and Prevention; FDA: US Food and Drug Administration; IV Rehydration: Intravenous rehydration; NVSN: New Vaccine Surveillance Network; PCV: *Porcine circovirus*; VRBPAC: Vaccines and Related Biological Products Advisory Committee.

## Pre-publication history

The pre-publication history for this paper can be accessed here:

http://www.biomedcentral.com/1471-2431/11/83/prepub
